# Genomic epidemiology of *Salmonella* Typhi in Central Division, Fiji, 2012 to 2016

**DOI:** 10.1016/j.lanwpc.2022.100488

**Published:** 2022-06-16

**Authors:** Mark R. Davies, Sebastian Duchene, Mary Valcanis, Aaron P. Jenkins, Adam Jenney, Varanisese Rosa, Andrew J. Hayes, Aneley Getahun Strobel, Liam McIntyre, Jake A. Lacey, Elizabeth J. Klemm, Vanessa K. Wong, Aalisha Sahukhan, Helen Thomson, Andrew Page, Dianna Hocking, Nancy Wang, Litia Tudravu, Eric Rafai, Gordon Dougan, Benjamin P. Howden, John A. Crump, Kim Mulholland, Richard A. Strugnell

**Affiliations:** a Department of Microbiology and Immunology, The University of Melbourne at the Peter Doherty Institute for Infection and Immunity, Victoria, Australia; b Microbiological Diagnostic Unit Public Health Laboratory, Department of Microbiology and Immunology, The University of Melbourne at the Peter Doherty Institute for Infection and Immunity, Melbourne, Australia; c Centre for Ecosystem Management, Edith Cowan University, Western Australia.; d School of Public Health, University of Sydney, Sydney, NSW, Australia; e New Vaccines Group, Murdoch Children's Research Institute, Victoria, Australia; f College of Medicine, Nursing and Health Sciences, Fiji National University, Suva, Fiji; g Fiji Centre for Disease Control, Fiji Ministry of Health, Suva, Fiji; h Department of Infectious Diseases, The University of Melbourne at the Peter Doherty Institute of Infection and Immunity, Victoria, Australia; i Wellcome Sanger Institute, Wellcome Genome Campus, Hinxton, Cambridge, United Kingdom; j Department of Medicine, University of Cambridge, Cambridge, United Kingdom; k Quadram Institute Bioscience, Norwich Research Park, Norfolk, United Kingdom; l Colonial War Memorial Hospital, Suva, Fiji; m Fiji Ministry of Health and Medical Services, Suva, Fiji; n Centre for International Health, Otago Medical School, University of Otago, Dunedin, New Zealand; o Department of Infectious Disease Epidemiology, London School of Hygiene and Tropical Medicine, London, United Kingdom

**Keywords:** Salmonella Typhi, Typhoid fever, Fiji, Outbreak, Public health, Phylodynamics, Bayesian, Phylogeny, Population genomics

## Abstract

**Background:**

Typhoid fever is endemic in some Pacific Island Countries including Fiji and Samoa yet genomic surveillance is not routine in such settings. Previous studies suggested imports of the global H58 clade of *Salmonella enterica* var Typhi (*Salmonella* Typhi) contribute to disease in these countries which, given the MDR potential of H58, does not auger well for treatment. The objective of the study was to define the genomic epidemiology of *Salmonella* Typhi in Fiji.

**Methods:**

Genomic sequencing approaches were implemented to study the distribution of 255 *Salmonella* Typhi isolates from the Central Division of Fiji. We augmented epidemiological surveillance and Bayesian phylogenomic approaches with a multi-year typhoid case-control study to define geospatial patterns among typhoid cases.

**Findings:**

Genomic analyses showed *Salmonella* Typhi from Fiji resolved into 2 non-H58 genotypes with isolates from the two dominant ethnic groups, the Indigenous (iTaukei) and non-iTaukei genetically indistinguishable. Low rates of international importation of clones was observed and overall, there were very low levels an antibiotic resistance within the endemic Fijian typhoid genotypes. Genomic epidemiological investigations were able to identify previously unlinked case clusters. Bayesian phylodynamic analyses suggested that genomic variation within the larger endemic *Salmonella* Typhi genotype expanded at discreet times, then contracted.

**Interpretation:**

Cyclones and flooding drove ‘waves’ of typhoid outbreaks in Fiji which, through population aggregation, poor sanitation and water safety, and then mobility of the population, spread clones more widely. Minimal international importations of new typhoid clones suggest that targeted local intervention strategies may be useful in controlling endemic typhoid infection. These findings add to our understanding of typhoid transmission networks in an endemic island country with broad implications, particularly across Pacific Island Countries.

**Funding:**

This work was supported by the Coalition Against Typhoid through the Bill and Melinda Gates Foundation [grant number OPP1017518], the Victorian Government, the National Health and Medical Research Council Australia, the Australian Research Council, and the Fiji Ministry of Health and Medical Services.

**Research in context**

**Evidence before the study**

Typhoid fever, caused by the human pathogen *Salmonella* Typhi, is endemic in some Pacific Island countries including Fiji. There is a lack of understanding of the population structure of *Salmonella* Typhi in Pacific Island Countries. We searched PubMed (manuscripts prior to September 2021) with no language restrictions using the terms “typhoid” and “Fiji” and“genomics”. The one resulting study examined the global dissemination of the single *Salmonella* Typhi clone, termed H58, throughout the world which included sporadic samples from Fiji and neighbouring countries. A second *Salmonella* Typhi genotyping paper from our extended research team also included sporadic genome samples from Fiji, yet these were restricted to ad hoc collections, often travellers, that lacked allied metadata. No study has undertaken a detailed longitudinal genomic epidemiology assessment to characterise the evolutionary dynamics of typhoid clones in a Pacific Island Country such as Fiji.

**Added value of this study**

In this retrospective, genomic epidemiology study, we present a spatiotemporal analysis of typhoid cases from the Central Division of Fiji from 2012 to 2016. We identify that 99% of typhoid cases in Fiji are driven by two closely related *Salmonella* Typhi genotypes (genotype 4.2.1 and 4.2.2) with extended regional analyses indicating that Pacific Island countries appear to have their own genetically distinct typhoid genotypes. Through coupling of genomics and epidemiology, we define genome clusters and track their spread associated with documented public health outbreaks. Phylogenomic analyses revealed ‘waves’ of typhoid cases were associated with population displacement through major events such as cyclones.

**Implications of all the available evidence**

Our study highlights that typhoid in Fiji is an endemic situation driven through evolution and transmission of regional *Salmonella* Typhi clones, rather than driven through frequent importation of new clonal variants. While the continuing high burden of typhoid is concerning, genomic surveillance can shed new light into the transmission chains of outbreak cases, which in the future, can inform the way public health agencies characterise typhoid clones and develop public health interventions.

Alt-text: Unlabelled box

## Introduction

The Republic of Fiji consists of 332 islands, and approximately 890,000 people,^1^ located in the tropical south Pacific. The largest island, Viti Levu, is volcanic in origin and the eastern slopes receive approximately 3 metres of rain per year. The tholeiitic basalt geology of Viti Levu mitigates against improved sanitation, and typhoid control will depend on better understanding of transmission, improved sampling, increased diagnosis, contact tracing, and ultimately, also vaccination. The origins of typhoid fever in Fiji are unclear but the reported incidence increased significantly in the mid-2000s and the trend for elevated numbers of culture-confirmed cases has continued.^2^, ^3^, ^4^, ^5^

A seroepidemiology study in 2014 suggested that 32.2% of the Fijian population have detectable and often high levels of anti-Vi antibodies, typically a marker of recurrent typhoid infections.^6^ Against this background of endemicity, focal outbreaks associated with major tropical storms and cyclones, increasingly attributed to climate change and the warming of nearby oceans, occur.^7^ Transmission remains somewhat enigmatic. The causitive bacterium for typhoid fever, *Salmonella* Typhi, is considered monophyletic,^8^, ^9^, ^10^, ^11^ human host-restricted, is difficult to isolate from the environment, and is generally transmitted faecally-orally through contaminated food or water.^12^^,^^13^ Some individuals infected with *Salmonella* Typhi become asymptomatic carriers, often harbouring the bacterium in their gallbladder; and intermittently transmit the pathogen through contaminated feces.

Our study used bacterial genomics to address two important questions that will impact on the control of typhoid fever in Fiji. First, is the pathogen related to the dominant multi-drug resistant (MDR) global H58 clade of *Salmonella* Typhi^11^^,^^14^ and are there ongoing importations? Second, can genomics-based micro-epidemiology be used to help resolve the networks of transmission of typhoid fever in Fiji? The results will focus and enhance surveillance of typhoid fever in Fiji, and in the region, as a prelude to the introduction of the new conjugate typhoid vaccine.^15^

## Methods

### Isolates and genome sequencing

*Salmonella* Typhi clinical isolates from Central Division, Fiji were collected by the Colonial War Memorial Hospital, Suva during 2012 to 2016 (n = 255, Supplementary Table 1). 175 consenting individuals from a recent case-control study^16^ which ran from 27 January 2014 through 31 January 2017 were encompassed within this genomic epidemiology study. 240 isolates represent culture confirmed blood isolates. Ten stool culture isolates from public health reponses to localised typhoid outbreaks were also included. Isolates were coupled with allied epidemiological metadata where available including known epidemiological outbreak cases as undertaken by Fiji Centre for Disease Control and/or in conjunction with the recent case-control study.^16^ The households of 129 cases with 135 samples accounting for paired blood/stool samples were also geo-located with a hand-held GPS as part of contact tracing processes. By definition, a typhoid outbreak in Fiji is based on the 2010 national guidelines where 2 or more suspected or confirmed cases of typhoid fever are identified within 1 month in a new area/village.

Laboratory confirmed *Salmonella* Typhi isolates were sent to the Microbiological Diagnostic Unit - Public Health Laboratory of the University of Melbourne, Australia, for genome sequencing. DNA was extracted from a single colony using a QIAsymphony DSP virus pathogen kit (Qiagen). Genome sequencing was performed using Nextera XT (Illumina) on the NextSeq 500/550 platform using 150 paired-end reads (Doherty Applied Microbial Genomics, Melbourne).

### Complete genome sequence of endemic Fijian genotype 4.2.2

The complete genome of a Fijian endemic genotype 4.2.2 strain ERL072973 was sequenced on an Illumina HiSeq2500 in rapid run mode, as described previously,^11^ to produce two sets of paired ended short read data (ERR343279, ERR343374), with a read length of 100 bases. The sample was also sequenced on a PacBio RSII with 2 SMRT cells to produce two sets of long read data (ERR581097, ERR581107). A hybrid assembly was performed using *Unicycler* (v0.4.0)^17^ with the long and short read datasets. Corrected reads were subsequently generated using the PacBio SMRT analysis pipeline (v2.3.0) for the long read datasets. The *Unicycler* assembly and the corrected reads were provided to *circlator* (v1.4.0)^18^ which produced a single circularised chromosome of 4,782,100 bases and is deposited in Genbank database under the identifier LT904777.

### Mapping and SNP calling

To determine phylogenetic structure in the Fijian 4.2.1 and 4.2.2 subclades, Illumina reads from 251 genotype 4.2 isolates (2012-2016) were mapped to *Salmonella* Typhi strain ERL072973 (genotype 4.2.2, Genbank id LT904777.2) using BWA-MEM2 as part of *snippy* v4.6.0 (github/tseemann/snippy). A total of 389 core genome SNPs were identified of which 190 were parsimony-informative. A similar process was undertaken to examine the structure of *Salmonella* Typhi genotype 4.2 within an extended database of 368 Fijian genotype 4.2 genome sequences representing 1981 - 2016^10^ (Supplementary Table 2). The extended 4.2.1/4.2.2 genotypic database was mapped to *Salmonella* Typhi strain ERL072973 (genotype 4.2.2) using BWA-MEM2 as part of *snippy* v4.6.0 (github/tseemann/snippy). A total of 766 SNP and indel events were identified in the mapping using *FreeBayes* v.1.3.1^19^ and functional annotations of SNPs called using *SnpEff* v4.3t^20^ as part of *snippy* (Supplementary Table 3). For core genome determination and tree building mobile genetic elements and genomic regions of irregular SNP density were identified in the reference genome and the isolate core genome alignment using *Gubbins* v2.4.1.^21^ All low complexity mapping regions, high SNP density regions and mobile genetic elements were then excised from the alignment resulting in a 4,570,660 bp core genome alignment consisting of a total of 492 core genome SNPs, including 156 parsimony informative sites and 336 singleton sites.

To put the Fijian case-control genome sequencing within a global context, a global database of genome sequences was collated (Supplementary Table 4) which is an extension of initial global framework^10^ with the addition of published datasets from India, Nigeria, and Uganda.^22^, ^23^, ^24^ To reconstruct the global *Salmonella* Typhi phylogeny, the Fiji sequence reads were mapped to the global CT18 reference genome (AL513382) using the same parameters as previously described.^10^ SNPs in repeat regions, prophage regions, and plasmid sequences (∼354 kb) were excluded for phylogenetic analyses. This global framework of 2,643 isolates (Supplementary Table 4) resulted in a set of 26,803 chromosomal SNPs (in respect to CT18 reference genome) within an alignment of length 4,275,037 bp.

### Genome assemblies, genotyping and gene screening

De novo draft genome assemblies were generated from Illumina short reads using SPAdes v3.14.1. The draft genomes were screened for known antimicrobial resistance genes using ABRicate v 1.0.1 (https://github.com/tseemann/abricate) using a 90% nucleotide similarity and length Blast threshold. In silico screens for chromosomal SNPs, indels and genes were screened using the pathogenwatch AMR prediction pipeline v2.4.12.^25^ Carriage of the typhoid toxin^26^ was determined at a 90% BlastN length/similarity cut-off using screen_assembly3.py.^27^ Genotyping of the genome sequences was determined using the genotyphi framework (https://github.com/katholt/genotyphi).^10^

### Phylogenetic and phylodynamic analyses

Phylogenetic relationships were inferred by both maximum-likelihood and Bayesian inference, using the single nucleotide polymorphism (SNP) alignment. Consensus SNP alignments were used to build a maximum-likelihood tree with IQ-TREE v1.6.11.^28^ A general time-reversible model with a gamma distribution to describe among-site rate heterogeneity was selected (GTR+F+Γ) for analysis in IQ-TREE, and with 1000 ultra-fast nonparametric bootstrap replicates to assess topological uncertainty.

Molecular clock phylogenetic analysis was conducted with a Bayesian approach in BEAST v1.10^29^ using an GTR+Γ substitution model with an uncorrelated lognormal relaxed clock model (UCLN). The UCLN model was selected over a strict clock (SC) model after inspecting the coefficient of rate variation (standard deviation of branch rates divided by their mean) in the UCLN model, an informal measure of clocklike behaviour that has been shown to have similar performance to methods based on marginal likelihoods.^30^ However, we note that the estimates from both molecular clock models had overlapping posterior densities. We set the Skygrid tree prior, a semi-parametric model where population size is estimated at different coalescent intervals but large demographic changes are penalised.^31^ To sample from the posterior distribution a Markov chain Monte Carlo was run for 10^8^ iterations with sampling every 10^4^ iterations. The first 10% of steps from the chain were discarded as burn-in. Sufficient sampling was assessed from the stationary distribution by verifying that the effective sample size for key parameters was at least 200. Convergence was assessed by repeating the analyses and ensuring that the posterior samples matched.

To estimate the infected population size, we fit a constant exponential growth coalescent model.^32^ Although this parametric model does not have the flexibility of skyline methods, it has explicit assumptions  and the estimates of demographic parameters are straight-forward to interpret. In particular, here we assume that we can approximate the average population trajectory with an exponential function and that the duration of infection of about 7 days. We estimated an infected population size at the time of collection of the most recent sample of around 2000 individuals (posterior mode = 2049.739; 95% credible interval: 1490.442 - 2777.179). Importantly, our estimate for this parameter was robust to the prior specified in the model (Supplementary Figure 6).

### Whole genome clustering

To determine whole genome clusters, the filtered core SNP alignment of the 251 genomes was loaded into R and pairwise SNP distances calculated using the dist.dna function in *ape*^33^ (model=”N”). Hierachical clustering of the isolates was performed using the *hclust* function as part of the R ‘stats’ package and clusters were filtered using a threshold of 2 SNPs for cluster membership using *cutree*. The 2 SNP threshold was defined based on the SNP accumulation rate across the whole genome in our study (∼0.6 SNPs per year). Clustering at a 0 SNP threshold was also performed to help link identical clones within clusters. Clusters were plotted if containing greater than 2 isolates at a maximum 2 SNP threshold. Genome clusters were also plotted against the phylogenetic tree using *ggtree.*^34^

### Role of the Funding Source

This study was supported by National Health and Medical Research Council of Australia (NHMRC) Program Grants ID606788 and ID1092262 and by the Coalition Against Typhoid through the Bill and Melinda Gates Foundation [OPP1017518]. The Colonial War Memorial Hospital is supported by the Fiji Ministry of Health and Medical Services. The Microbiological Diagnostic Unit Public Health Laboratory is funded by the Victorian Government. MRD is supported by a University of Melbourne CR Roper Fellowship. BPH is supported by an NHMRC Investigator Grant (GNT1196103). SD is supported by an Australian Research Council Discovery Early Career Researcher Award (DE190100805). GD was supported by an NIHR BRC AMR award and funding from UKRI Vaccine Hub, UKRI AMR, STRATAA and TyVac Gates. The funders had no role in study design, data analysis, data interpretation, writing of this report or decision to submit the paper for publication.

## Results

### Dominance of non-H58 *Salmonella* Typhi genotypes in Fiji

In this study, *Salmonella* Typhi was isolated from blood and/or stool of suspected patients in the Central Division of Fiji and the disease diagnosed using standard microbiology and *Salmonella* Typhi-specific Vi capsular antiserum. The genomes of 255 *Salmonella* Typhi isolates were initially analysed (Supplementary Table 1), and the patient demographics are shown in Table 1. The isolates were predominantly collected during a case-control study of typhoid fever in the Central Division of Fiji from 2012 to 2016.^16^ The households of 129 cases were also geo-located with a hand-held global positioning system (GPS).

**Table 1:** Demographics of patients contributing isolates, Central Division, Fiji, 2012-2016.

Ethnicity		Gender		Age (years)	Isolation Site		*S*. Typhi strain genotype+			
		Female	Male	Median (IQR)	Blood	Stool	3.5	4.2.1	4.2.2	4.3.1
FID	7	4 (57%)	2 (29%)	14 (13-19.5)	6	0	0	1	5	1
IT	237	118 (50%)	117 (50%)	27 (16-39)	226	10	2	74	161	0
FOD	1	1	0	3	1	0	1	0	0	0

Acronyms: FID, Fijian of Indian Descent; IT, iTaukei (Indigenous Fijian); FOD, Fijian of other descent; IQR, interquartile range.

+ Footnotes: Number of isolates belonging to the respective genotyphi genotype.

Sequencing reads were mapped to the archetypical *Salmonella* Typhi reference genome CT18 (accession AL513382).^35^ Applying the Genotyphi typing nomenclature,^10^ 251 (98.4%) of the 255 isolates were genotype 4.2, 3 (1.2%) isolates were genotype 3.5, and 1 (0.4%) isolate was from genotype 4.3.1 (H58). The two major genotypic subclades, 4.2.1 (75 isolates) and 4.2.2 (176 isolates) were detected over multiple years, with no obvious variation in relative frequency (Supplementary Figure 1A). The single H58-clade isolate was obtained from a patient who was diagnosed with typhoid fever soon after returning from elective surgery in India, in 2014.

**Figure 1:**
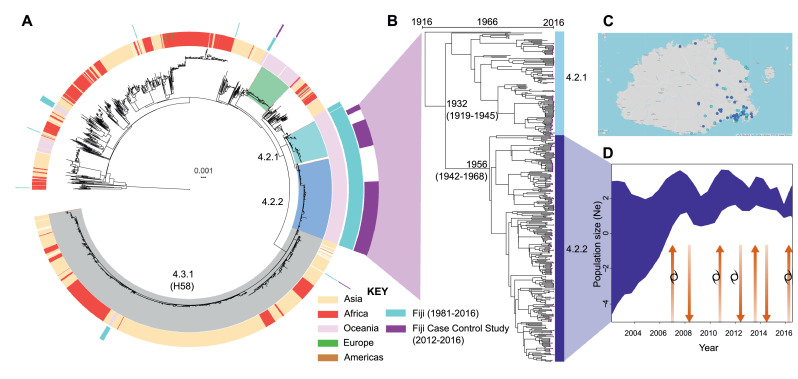
Population dynamics of typhoid fever in Fiji. A) Maximum-likelihood phylogeny of 2,643 global *Salmonella* Typhi mapped to *Salmonella* Typhi CT18 and rooted to *Salmonella* Paratyphi A. Isolates belonging to *Salmonella* Typhi genotype 4.2.1, 4.2.2 and 4.3.1 (H58) are shaded. Rings (from inner to outer) refer to; geographical region of global *Salmonella* Typhi; all published Fijian genotypes (1981-2016, aqua); and Central Division Fiji isolates characterised in this study (2012-2016, purple). B) Bayesian temporal population structure of 366 Fijian endemic genotype 4.2.1 and 4.2.2 obtained between 1981 to 2016. Purple tips reflect location of the 2012-2016 isolates. C) Geographical distribution of the 2 major Fijian genotypes in the Central Division based on GPS coordinates associated with 128 isolates. D) Bayesian population dynamics of Fiji genotype 4.2.2 inferred using a Skygrid model, such that the y-axis represents genetic diversity and is proportional to infected population size. The arrows indicate ‘waves’ of sub-clonal expansion and contraction (arrows) in the context of time. Occurence of major cyclones (Daman, December 2007; Tomas, March 2010; Evan, December 2012; Winston; February 2016) in Fiji are indicated by the cyclone symbol.

The genomics of the isolates were placed into a global context by mapping the data against a framework of 2,466 *Salmonella* Typhi isolates from 6 continents.^10^^,^^14^ Within this extended framework, isolates from the Fijian case-control study isolates clustered into three main ancestral genotypes (Figure 1A, purple ring). Intriguingly, genotype 4.2 provided 389 (99%) of 391 clinical isolates from Fiji, indicating that genotype 4.2 represents a geographically constrained genotype that is endemic to the Fijian islands. The remaining two genotype 4.2 isolates were from Tonga.^10^ The three Fijian case-control study genotype 3.5 isolates clustered with isolates that are ancestral to genotype 3.5.4 that accounted for 105 (99%) of 106 Samoan isolates,^10^ suggesting that some strains may circulate between Pacific Island Countries, even though the Pacific Countries appear to have their own genetically distinct genotypes (Supplementary Figure 2).

### Low rates of multi-drug resistant *Salmonella* Typhi in Fiji

Given the global rise in ciprofloxacin and ceftriaxone resistance in *Salmonella* Typhi^11^^,^^14^ we screened genome assemblies of Fijian isolates for the presence of resistance alleles. Two isolates carried mutations in DNA gyrase (*gyrA* (S83F)), which are linked with ciprofloxacin resistance.^36^ These isolates were collected in 2014 and 2015 and resistance was phenotypically confirmed. All the isolates collected were positive for the typhoid toxin,^26^ although re-analyses of the historical Fijian isolate database^10^ identified one isolate that appeared to be toxin-negative. Unfortunately, the case files for this patient were not accessible.

### Phylogenomic ‘waves’ of endemic sub-clones associate with population displacement

To identify specific genetic markers of the Fijian endemic genotype, we determined the genome sequence of an ancestral 4.2.2 *Salmonella* Typhi strain collected in 2007 from Fiji (ERL072973). In order to define the phylogenetic structure of the Fiji-endemic *Salmonella* Typhi, we used Bayesian modelling approaches to determine the evolutionary relationship of genotype 4.2.1/4.2.2 isolates. First, we mapped the extended 4.2.1/4.2.2 genotypic database (n=368; representing isolates collected between 1981-2016) to the newly generated genotype 4.2.2 reference genome (ERL072973). Application of Bayesian molecular clock analyses revealed that the 4.2.1/4.2.2 sub-lineages likely diverged from a common ancestor circa 1970s (95% highest posterior density (HPD) 1958-1978, Figure 1B) with 4.2.2 being the most clinically prevalent (253 of 368, 69%). Population dynamics of the predominant 4.2.2 genotype revealed that the effective population size, a parameter proportional to the number of infected cases, of these clones were not constant, with 4 ‘waves’ of genomic variation, followed by contraction in 2007, 2010, 2012, and 2016, with no clear seasonal periodicity (Figure 1D). Application of an exponential growth coalescent model to the dataset^32^ allows for a crude estimation of the putative number of typhoid infections at the date of the last collected genome sample. We estimated an infected population size at the time of the most recent sample is approximately 2,000 individuals (HPD: 1490–2777) in the Central Division of Fiji. Importantly, as this model assumes that population dynamics can be approximated by a deterministic exponential function, estimates of population size should be interpreted with caution. The overall rate of evolution of genotype 4.2.2 is 1.27×10^−7^subs/site/year (HPD: 9.48×10^−8^-1.69×10^−7^) which is comparable to the published rate of 1.7×10^−7^ (CI; 1.1×10^−7^-2.2×10^−7^) for 4.3.1 (H58)^37^ (Supplementary Figure 3).

Periods where increased genomic variation occurred were marked by outbreaks associated with cyclones (Figure 1D) and genomic epidemiology suggesting that cases in the population displaced by cyclones resulted in spread of *Salmonella* Typhi clones into other geographical settings. Fiji was impacted by Cyclone Daman in 2007, Cyclone Tomas in 2010, Cyclone Evan in 2012, Cyclone Winston in 2016. The cyclones damaged housing and/or produced rain depressions which inundated sanitation facilities, often affecting water supplies, an underlying risk factor resulting in increased *Salmonella* Typhi exposure in Fiji.^38^^,^^39^ It is tempting to speculate that increased cyclonic intensity, driven by climate change,^40^ is accelerating the evolution of *Salmonella* Typhi in Fiji.

### Co-circulation and transmission dynamics of genomic clusters

To determine the phylogeographical context of the typhoid fever cases we examined, GPS coordinates were obtained for 128 (50.2%) of 255 typhoid isolates. To test the hypothesis that the two major subclades were geographically dispersed, maps were drawn for Fijian genotypes 4.2.1 and 4.2.2. Phylogeographical analyses suggests that there is no geographical structure of the two 4.2 sublineages; both are equally distributed (Figure 1C) and that there is no apparent geographical restriction to the movement of these two clinically relevant genotypes within the Central Division of Fiji.

**Figure 2:**
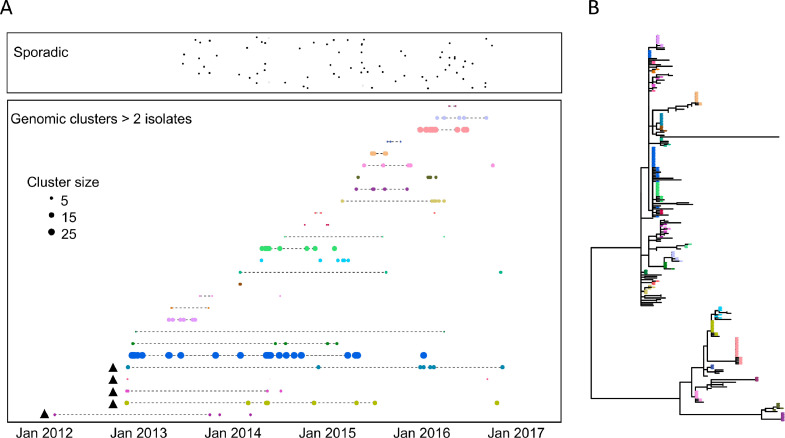
Timeline of *Salmonella* Typhi genomic clusters in Fiji. A) Each typhoid case is represented by a single dot and are classified as being sporadic single genomic cases (top box) or belonging to 27 genomic clusters (defined as containing 3 or more isolates related by <=2 core chromosomal SNPs). Cases are plotted by date of sampling (x-axis) and genomic cluster (y-axis) with size of cluster relative to total number of isolates in the cluster. Dotted lines refer to exact clones (no SNP differences). Triangle refer to isolates collected before case-control study where date of collection was estimated. B) Phylogenetic relationship of 251 Central Division isolates built from 252 SNPs and color coded by genomic cluster represented in (A). Multiple genomic outbreak clusters are represented at any one time some of which can persist for several years, some of which can spread between geographical regions (Supplementary Figure 4).

**Figure 3:**
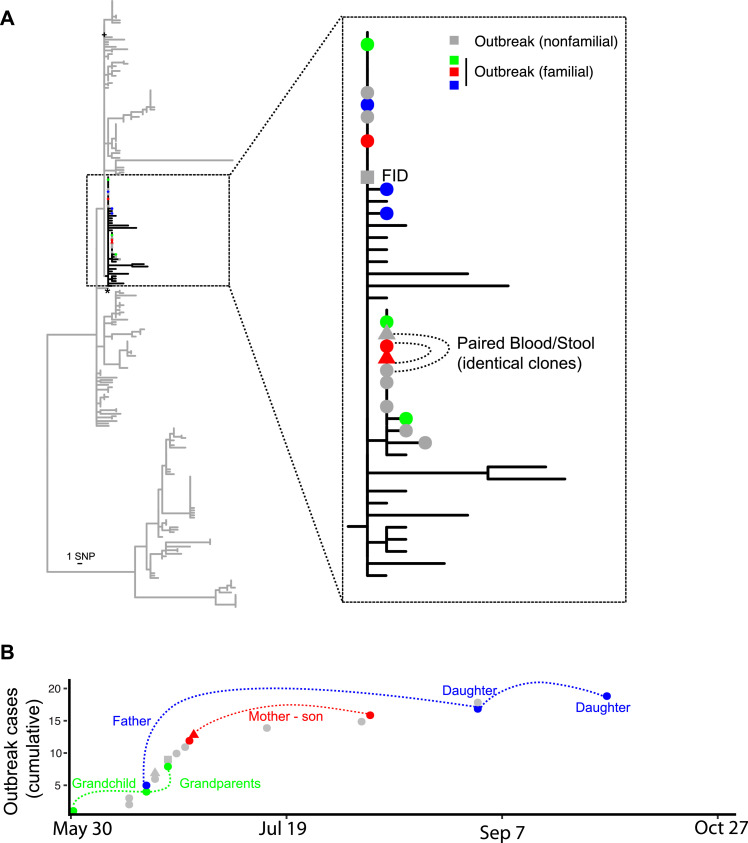
Epidemiological investigation of a prolonged *Salmonella* Typhi outbreak at Wailoku village, Central Division, Fiji. (A) Maximum-likelihood phylogenetic tree of all 251 genotype 4.2 *Salmonella* Typhi cases from Central Division Fiji, with insert showing zoomed in relationship of cases associated with the Wailoku outbreak. Colored circles indicate familial transmission chains (nonfamilial outbreak strains in grey). Stool samples are indicated by a triangle. Square sample is from a Fijian of Indian Descent (FID) while all remaining outbreak isolates are from iTaukei Fijians. (B) Epidemiological curve of Wailoku village outbreak case numbers (Y-axis being cumulative cases for which genome sequence data was obtained). Colors and connecting lines refer to familial epidemiological associations derived from contact tracing of clinical cases.

The phylogeographic study suggested that many of the diagnosed typhoid infections in our study resulted from common source outbreaks, with samples drawn from a particular location tending to be clustered in the phylogenetic tree. A total of 27 genomic clusters (defined by ≤ 2 core chromosomal SNPs, > 2 isolates) were identified comprising 71% (177/251) of reported cases with a median of 8 cases per cluster (range 3 to 27 cases, IQR 5 – 18 cases) (Figure 2). The median duration of persistence of a cluster was 335 days (range 2 to 1,445 days, IQR 161 to 771 days). Many of these genomic clusters were not geographically constrained (Supplementary Figure 4), consistent with phases of long infection quiescence followed by both local and regional transmission. These data indicate that multiple typhoid clones circulate at any one time, some of which can persist for multiple years in either within a human host or unsampled reservoir. Two major typhoid outbreaks were subjected to public health investigation by the Fiji Ministry of Health and Medical Services during the case-control study, one in Wailoku settlements associated with a commemorative gathering^41^ and another at Qelekuro associated with Cyclone Winston. These two outbreaks were genetically unrelated, but demonstrate the spread the clones through different regional settings after communal gatherings and/or displacement through climatic events (Supplementary Figure 5). We conducted a detailed analysis of cases in Wailoku settlements over a 5-month period in 2014 (Figure 3). The reported outbreak from May to June involved 22 cases, where there were 13 laboratory confirmed cases and 9 probable cases.^41^ Further cases were diagnosed in same settlement areas in August, September and October 2014. Genomics on the isolates obtained from the total 19 Wailoku patients revealed fewer than 3 SNP differences, suggesting that the infections identified in August, September and October were unlikely to be exogenous reintroductions (Figure 3). Case workers determined that an individual infected in October was the child of a patient diagnosed in early June. One patient, a Fijian of Indian descent was diagnosed on June 21, and genomics revealed that this patient was infected with the same outbreak clone. Genomic analysis of previous *Salmonella* Typhi isolates from Fiji suggest that the Wailoku isolate was present elsewhere in Fiji in 2013, and, therefore, that the clone was first introduced into population on May 17, 2014 at a large memorial gathering. These findings support the use of bacterial genomics to identify transmission networks of typhoid fever in disease endemic settings like Fiji.

## Discussion

Typhoid fever is endemic in some Pacific Island Countries^42^, ^43^, ^44^ including Fiji.^4^^,^^5^ In this study, we applied genomic epidemiological and Bayesian statistical approaches to shed new light on the population dynamics of *Salmonella* Typhi in the Central Division of Fiji. In contrast to genomic epidemiological investigations from typhoid endemic settings such as Asia and Africa where the disease is dominanted by the multi-drug resistant H58 clone of *Salmonella* Typhi,^9^, ^10^, ^11^^,^^14^^,^^16^^,^^22^, ^23^, ^24^, ^25^ typhoid in Fiji is driven a genetically distinct genotype (genotype 4.2 subclades). In further contrast to these extant disease endemic settings, very low levels (<1%) of Fijian typhoid isolates were resistant to antimicrobial agents including ciprofloxacin, conferred by DNA gyrase polymorphisms. Such resistance appeared sporadic and has not become more widely established, perhaps due to strict control of ciprofloxacin use in Fiji. We also observe that Pacific Countries appear to have their own genetically distinct *Salmonella* Typhi genotypes, with infrequent transmission detected, yet this requires broader surveillance networks.

Genomics cannot explain the incidence discrepancy between the two major Fijian ethnic groups, the iTaukei (or indigenous Fijians), and the Fijians of Indian Descent (FID), which show similar Vi seroprevalence.^6^ This shared seroprevalence is in stark contrast to the reported incidence of typhoid fever in the two communities, where infections in FID are rarely reported.^6^ Differences in proportion of infections that are symptomatic by ethnicity might be explained by early presentation and syndromic treatment, access to treatment, or host genetics. In the case-control study,^16^ there were 7 typhoid cases in FID; where the isolate was obtained, the bacteria were members of the same genotypes that were present in the iTaukei. One exception was the *Salmonella* Typhi isolate that was brought to Fiji by a FID who had travelled from India; this isolate was from the H58 clade. Typhoid fever in India, like many countries, is now dominated by the H58 clade.^24^ While previous global analyses revealed several Fijian importations of H58 *Salmonella* Typhi,^11^ the H58 genotype is yet to displace the endemic *Salmonella* Typhi in Fiji, suggesting that targeted local intervention strategies may be useful in controlling endemic typhoid infection.

The phylogenetic studies reveal the power of genomics to track outbreaks, both within villages and across regional settings. The genomics data suggest that, despite *Salmonella* Typhi having a monophyletic population structure, it is possible to definitively identify whether disease in a village is caused by single or multiple introductions. Through these investigations, multiple typhoid clones were identified to circulate at any one time, some of which can persist for multiple years in either within a human host or unsampled reservoir. Combined with anti-Vi antibody assays, which can also be used to help identify carriers,^6^ genomics is an important tool in elucidating transmission pathways, identifying the causative genotypes in waves of localised outbreaks and informing intervention strategies.

## Contributors

RAS, KM, JAC, AJ, APJ, GD, MRD conceived the project. MRD, SD, RAS designed the experiments. MRD, SD, MV, APJ, AJ, VR, AJH, AGS, LM, JAL, AJK, AP performed the experimental protocols. MRD, SD, MV, AJH, AGS, LM, JAL, EJK, VKW, AS, HT, AP, DH, NW, LT, ER, GD, JBP, JAC, KM, RAS analyzed the experimental results. MRD, SD, AJH, RAS wrote the manuscript and all authors reviewed the manuscript. MRD and RAS contributed equally to this work.

## Data sharing statement

Illumina sequence reads and draft genome assemblies were deposited into the European Nucleotide Archive (Bioproject identifier PRJNA739044). Accession numbers for individual sequence reads are supplied in Supplementary Table 1.

## Editor note

The Lancet Group takes a neutral position with respect to territorial claims in published maps and institutional affiliations.

## Declaration of interests

The authors declare no competing interests.

## Appendix Supplementary materials

Image, application 1

Image, application 2

Image, application 3

Image, application 4

Image, application 5

Image, application 6

## References

[1] FBOS. Population and housing census 2017. *Suva, Fiji Islands* 2018.

[2] Kumar SA, Jacob A, Enari M (2012). The incidence of typhoid fever in Fiji from 1995-2009. Fiji J Public Health.

[3] Dunn J, Pryor J, Saketa S (2005). Laboratory-based *Salmonella* surveillance in Fiji, 2004-2005. Pac Health Dialog.

[4] Thompson CN, Kama M, Acharya S (2014). Typhoid fever in Fiji: a reversible plague?. Trop Med Int Health.

[5] Getahun Strobel A, Parry CM, Crump JA (2019). A retrospective study of patients with blood culture-confirmed typhoid fever in Fiji during 2014-2015: epidemiology, clinical features, treatment and outcome. Trans R Soc Trop Med Hyg.

[6] Watson CH, Baker S, Lau CL (2017). A cross-sectional seroepidemiological survey of typhoid fever in Fiji. PLoS Negl Trop Dis.

[7] Walsh KJE, Camargo SJ, Knutson TR (2019). Tropical cyclones and climate change. *Trop Cycl Res Rev*.

[8] Kidgell C, Reichard U, Wain J (2002). *Salmonella* Typhi, the causative agent of typhoid fever, is approximately 50,000 years old. Infect Genet Evol.

[9] Roumagnac P, Weill FX, Dolecek C (2006). Evolutionary history of *Salmonella* Typhi. Science.

[10] Wong VK, Baker S, Connor TR (2016). An extended genotyping framework for *Salmonella enterica* serovar Typhi, the cause of human typhoid. Nat Commun.

[11] Wong VK, Baker S, Pickard DJ (2015). Phylogeographical analysis of the dominant multidrug-resistant H58 clade of *Salmonella* Typhi identifies inter- and intracontinental transmission events. Nat Genet.

[12] Parry CM, Hien TT, Dougan G, White NJ, Farrar JJ. (2002). Typhoid fever. N Engl J Med.

[13] Saha S, Tanmoy AM, Andrews JR (2019). Evaluating PCR-based detection of *Salmonella* Typhi and Paratyphi A in the environment as an enteric fever surveillance tool. Am J Trop Med Hyg.

[14] Klemm EJ, Shakoor S, Page AJ (2018). Emergence of an extensively drug-resistant *Salmonella enterica* serovar Typhi clone harboring a promiscuous plasmid encoding resistance to fluoroquinolones and third-generation cephalosporins. mBio.

[15] World Health Ogranisation (2019). Typhoid vaccines: WHO position paper, March 2018 - Recommendations. Vaccine.

[16] Prasad N, Jenkins AP, Naucukidi L (2018). Epidemiology and risk factors for typhoid fever in Central Division, Fiji, 2014-2017: a case-control study. PLoS Negl Trop Dis.

[17] Wick RR, Judd LM, Gorrie CL, Holt KE. (2017). Unicycler: Resolving bacterial genome assemblies from short and long sequencing reads. PLoS Comput Biol.

[18] Hunt M, Silva ND, Otto TD, Parkhill J, Keane JA, Harris SR. (2015). Circlator: automated circularization of genome assemblies using long sequencing reads. Genome Biol.

[19] Garrison E, Marth G. Haplotype-based variant detection from short-read sequencing. arXiv:12073907 *[q-bioGN]*2012.

[20] Cingolani P, Platts A, Wang le L (2012). A program for annotating and predicting the effects of single nucleotide polymorphisms, SnpEff: SNPs in the genome of Drosophila melanogaster strain w1118; iso-2; iso-3. *Fly*.

[21] Croucher NJ, Page AJ, Connor TR (2015). Rapid phylogenetic analysis of large samples of recombinant bacterial whole genome sequences using Gubbins. Nucleic Acids Res.

[22] International Typhoid Consortium, Wong VK, Holt KE (2016). Molecular surveillance identifies multiple transmissions of typhoid in West Africa. PLoS Negl Trop Dis.

[23] Park SE, Pham DT, Boinett C (2018). The phylogeography and incidence of multi-drug resistant typhoid fever in sub-Saharan Africa. Nat Commun.

[24] Pragasam AK, Pickard D, Wong V (2020). Phylogenetic analysis indicates a longer term presence of the globally distributed H58 haplotype of *Salmonella* Typhi in Southern India. Clin Infect Dis.

[25] Argimon S, Yeats CA, Goater RJ (2021). A global resource for genomic predictions of antimicrobial resistance and surveillance of *Salmonella* Typhi at pathogenwatch. Nat Commun.

[26] Song J, Gao X, Galan JE. (2013). Structure and function of the *Salmonella* Typhi chimaeric A(2)B(5) typhoid toxin. Nature.

[27] Davies MR, McIntyre L, Mutreja A (2019). Atlas of group A streptococcal vaccine candidates compiled using large-scale comparative genomics. Nat Genet.

[28] Nguyen LT, Schmidt HA, von Haeseler A, Minh BQ (2015). IQ-TREE: a fast and effective stochastic algorithm for estimating maximum-likelihood phylogenies. Mol Biol Evol.

[29] Suchard MA, Lemey P, Baele G, Ayres DL, Drummond AJ, Rambaut A. (2018). Bayesian phylogenetic and phylodynamic data integration using BEAST 1.10. Virus Evol.

[30] Ho SY, Duchene S, Duchene D. (2015). Simulating and detecting autocorrelation of molecular evolutionary rates among lineages. Mol Ecol Resour.

[31] Ho SY, Shapiro B. (2011). Skyline-plot methods for estimating demographic history from nucleotide sequences. Mol Ecol Resour.

[32] Dearlove B, Wilson DJ. (2013). Coalescent inference for infectious disease: meta-analysis of hepatitis C. *Philos Trans R Soc Lond B*.

[33] Paradis E, Schliep K. (2019). *ape* 5.0: an environment for modern phylogenetics and evolutionary analyses in R. Bioinformatics.

[34] Yu G, Lam TT, Zhu H, Guan Y. (2018). Two methods for mapping and visualizing associated data on phylogeny using *ggtree*. Mol Biol Evol.

[35] Parkhill J, Dougan G, James KD (2001). Complete genome sequence of a multiple drug resistant *Salmonella enterica* serovar Typhi CT18. Nature.

[36] Eaves DJ, Randall L, Gray DT (2004). Prevalence of mutations within the quinolone resistance-determining region of *gyrA, gyrB, parC*, and *parE* and association with antibiotic resistance in quinolone-resistant *Salmonella enterica*. Antimicrob Agents Chemother.

[37] Duchene S, Holt KE, Weill FX (2016). Genome-scale rates of evolutionary change in bacteria. Microb Genom.

[38] de Alwis R, Watson C, Nikolay B (2018). Role of environmental factors in shaping spatial distribution of *Salmonella enterica* serovar Typhi, Fiji. Emerg Infect Dis.

[39] Scobie HM, Nilles E, Kama M (2014). Impact of a targeted typhoid vaccination campaign following cyclone Tomas, Republic of Fiji, 2010. Am J Trop Med Hyg.

[40] Knutson TR, McBride JL, Chan J (2010). Tropical cyclones and climate change. Nature Geoscience.

[41] Pablo R, Tuivuya N, Lawe L, Kalisogo K. (2015). Investigation on the typhoid outbreak in Wailoku, Tamavua, Suva. *Fiji J Public Health*.

[42] Clegg A. (1995). The role of the laboratory in the diagnosis and management of typhoid fever. PNG Med J.

[43] Sikorski MJ, Desai SN, Tupua S (2020). Tenacious endemic typhoid fever in Samoa. Clin Infect Dis.

[44] GBD 2017 Typhoid and Paratyphoid Collaborators (2019). The global burden of typhoid and paratyphoid fevers: a systematic analysis for the Global Burden of Disease Study 2017. Lancet Infect Dis.

