# Supplementary material for: Genomic epidemiology of Salmonella Typhi in Central Division, Fiji, 2012 to 2016
Source: Lancet Reg Health West Pac. 2022 Jun 16;24:100488. doi: 10.1016/j.lanwpc.2022.100488 (PMC9234096; doi:10.1016/j.lanwpc.2022.100488)
Supplement: Supplementary file 2 [file mmc2.pdf]

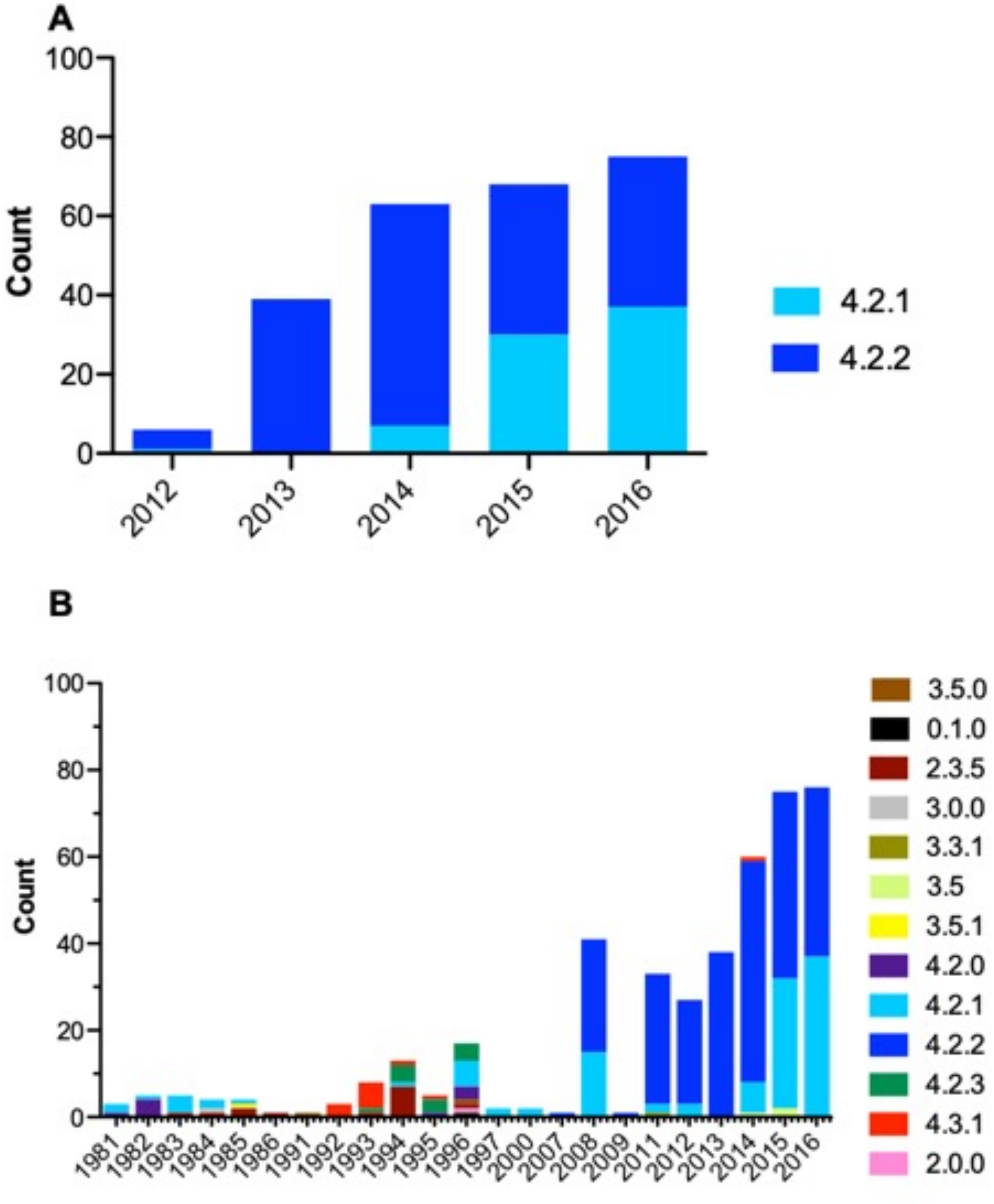

Supplementary figure 1. Frequency of *Salmonella* Typhi genotypes in Fiji during (A) Central Division case-control study (2012-2016, n = 251) and (B) within the global typhoid framework (1981-2016, n = 363). Colors refer to different genotypes as assigned by the genotyphi nomenclature.

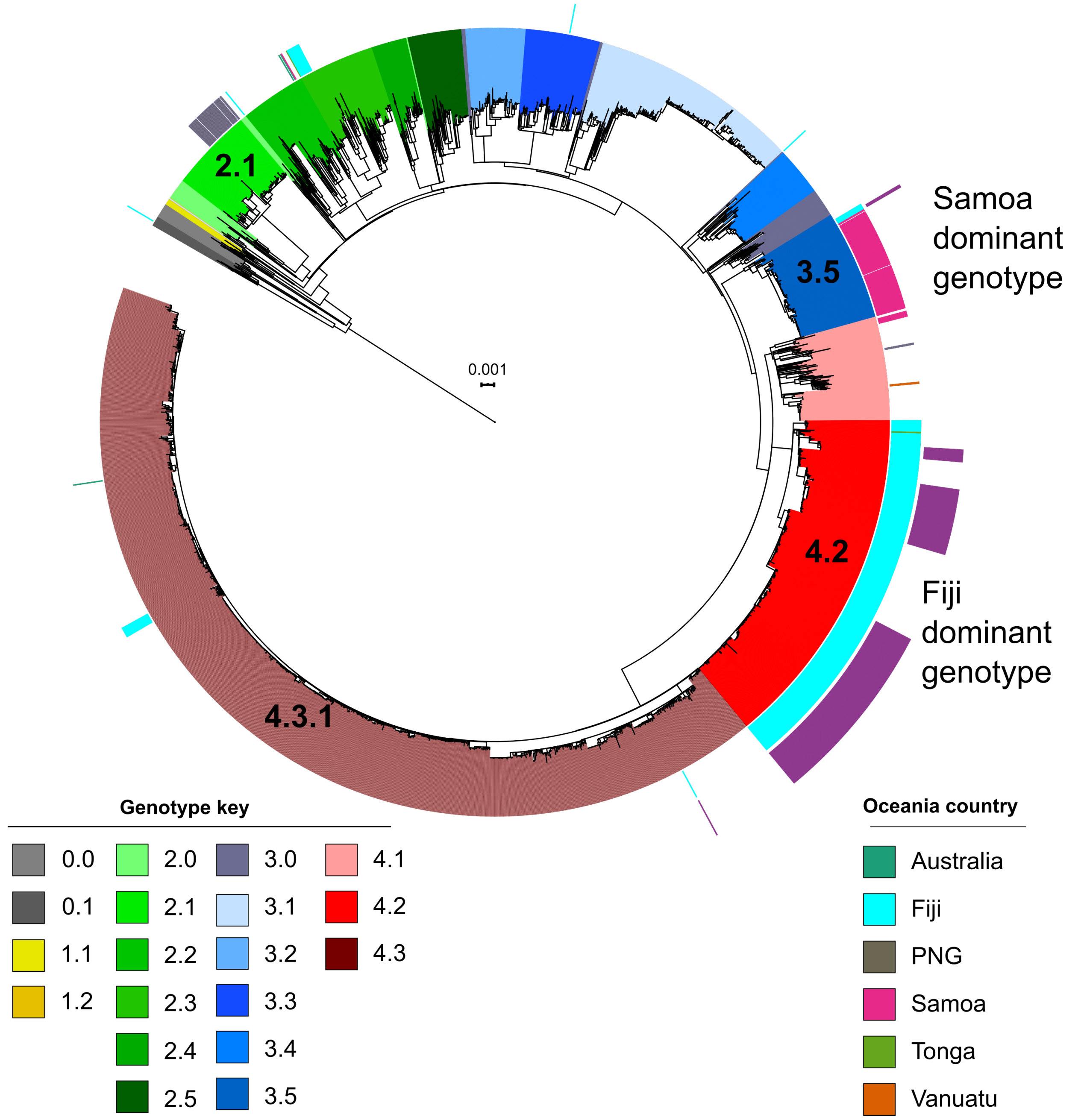

Supplementary figure 2. Distribution of *Salmonella* Typhi genotypes isolated from the Oceanic region within the global framework. Major clades are color coded by genotyphi nomenclature. Selected clades are annotated. Inner ring indicates isolates associated with countries from the oceanic regions. 4.2 genotype (red) is dominated by isolates from Fiji while 3.5 (predominantly genotype 3.5.4) is dominated by isolates from Samoa. Outer ring refers to Isolates obtained from the Central Division case-control study (2012-2016).

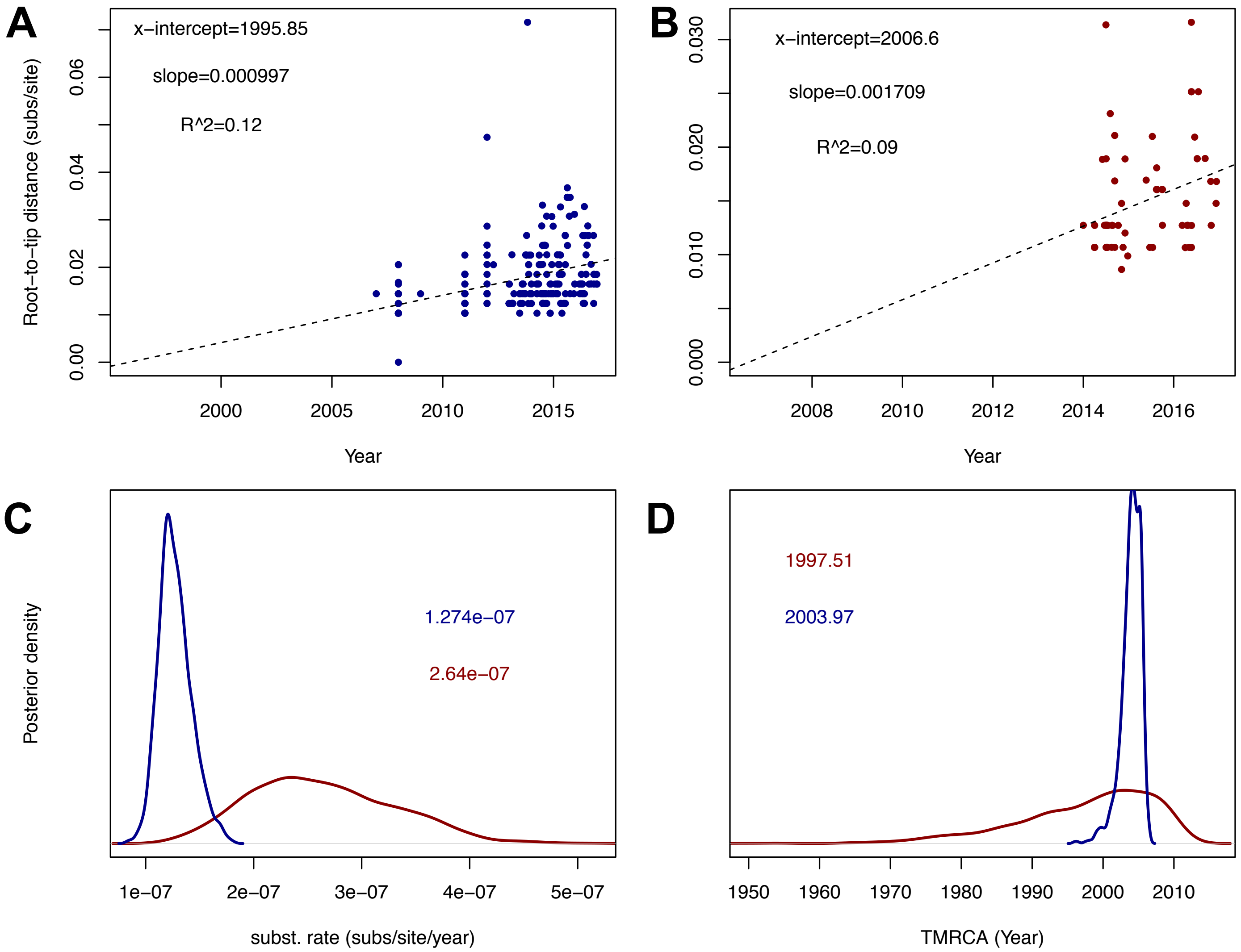

Supplementary figure 3. Root-to-tip regressions for the complete genotype 4.2 dataset (n = 251) (A) and only including samples with associated GPS coordinates (B). The slope and x-intercepts correspond to crude estimates of the evolutionary rate and time to the most recent common ancestor, respectively, while the R^2 is a measure of clocklike behavior. Panels (C) and (D) show the posterior densities of the evolutionary rate and time to the most recent common ancestor, respectively. The blue density represents the complete data set, while that in red is for only samples with associated GPS coordinates.

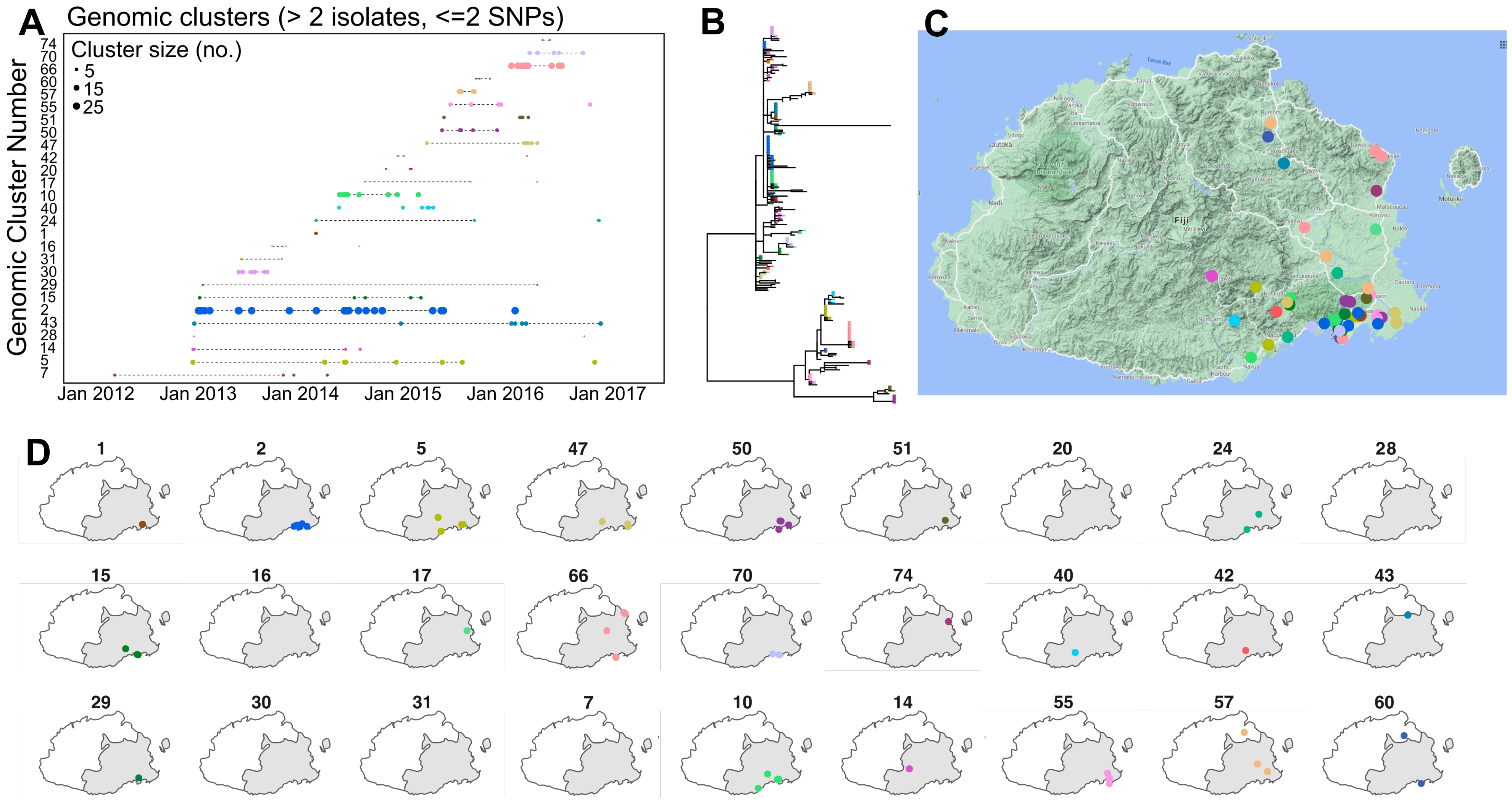

Supplementary figure 4. Geographical spread of *Salmonella* Typhi genomic clusters in Central Division, Fiji. A) Each typhoid case is represented by a single dot and are classified as being sporadic single genomic cases (top box) or belonging to 27 genomic clusters (defined as containing 3 or more isolates related by <=2 core chromosomal SNPs). Cases are plotted by date of sampling (x-axis) and genomic cluster (y-axis) with size of cluster relative to total number of isolates in the cluster. Dotted lines refer to exact clones (no SNP differences). B) Phylogenetic relationship of 251 Central Division isolates built from 252 SNPs and color coded by genomic cluster represented in (A). C) Geographical distribution of genomic clusters (A) based on GPS coordinates associated with 128 isolates. D) Individual plots of 27 genomic clusters as annotated in (A). Grey shading refers to the Central Division border. Of note, only cases with GPS coordinates were plotted.

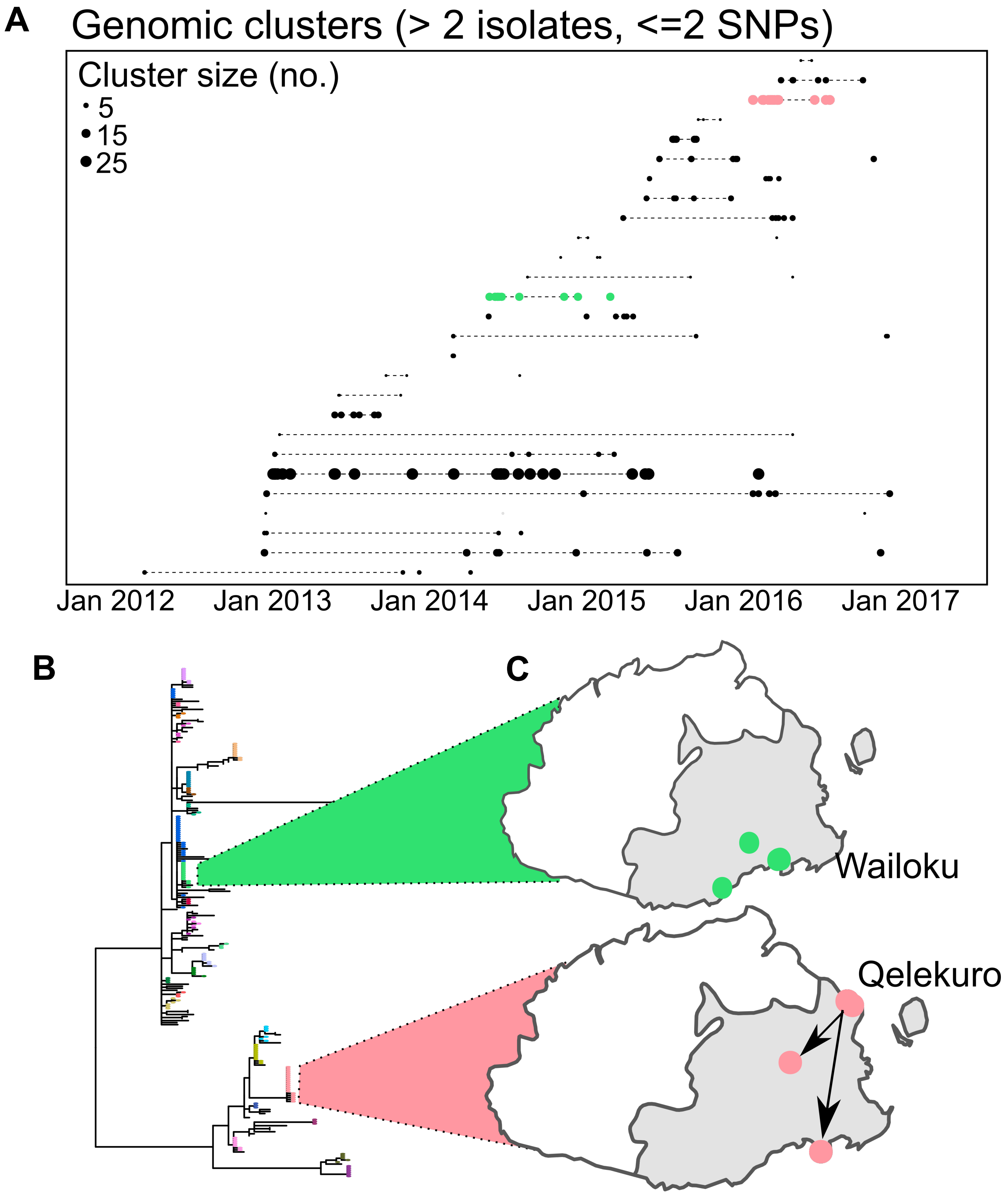

Supplementary figure 5. Genomic epidemiology of 2 community typhoid outbreaks notified to Fiji public health authorities during the course of the study. A) Timeline of 27 genomic clusters characterized in this study (defined as containing 3 or more isolates related by <=2 core chromosomal SNPs). Cases are plotted by date of sampling (x-axis) and genomic cluster (y-axis) with size of cluster relative to total number of isolates in the cluster. Dotted lines refer to exact clones (no SNP differences). B) Phylogenetic relationship of 251 Central Division isolates built from 252 SNPs and color coded by genomic cluster represented in (A). C) Geographical location of outbreak cases derived from contact tracing and GPS plotting. Arrows refer to temporal spread of cases as identified through contact tracing as was associated with movement of individuals from Qelekuro to close contacts following displacement due to Cyclone Winston.

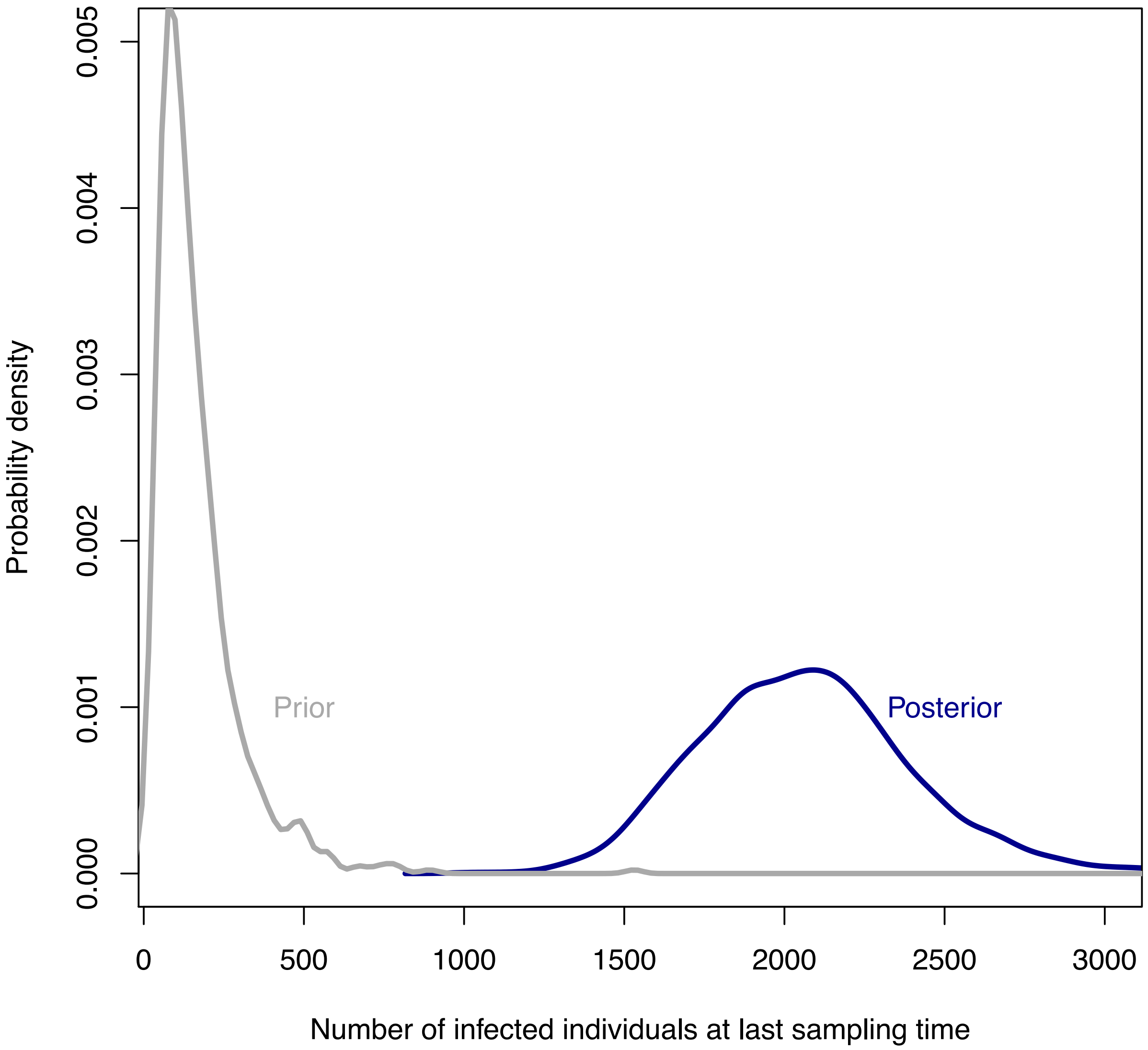

Supplementary figure 6. Prior and posterior distribution of the number of infected individuals at time of collection of the most recent sample. The grey line denotes the prior, such that it is the value for this parameter in absence of genomic data. The blue line corresponds to the posterior density, using the genomic data. The estimates were obtained using an exponential growth coalescent model that assumes that the average infected population size trajectory can be approximated using an exponential function and that the duration of infection is around 7 days.
